# Spontaneous coronary artery dissection presenting as an ischaemic stroke in a middle-aged man with anti-cardiolipin antibodies: a case report

**DOI:** 10.1186/1752-1947-4-94

**Published:** 2010-03-24

**Authors:** NS Rajendra, F Lim, N Shaukat

**Affiliations:** 1 Department of Cardiology, Kettering General Hospital, Rothwell Road, Kettering NN16 8UZ, UK

## Abstract

**Introduction:**

Cerebrovascular disease is a major cause of mortality and morbidity worldwide. Ischemic stroke is the most common manifestation, encompassing a wide variety of causative mechanisms. We present the case of a middle-aged male patient with spontaneous coronary artery dissection in the presence of anti-cardiolipin antibodies, leading to left ventricular thrombus and presenting with stroke.

**Case presentation:**

A 56-year-old Caucasian man presented with dysarthria and right-sided weakness. There was a history of chest pain with autonomic symptoms four days earlier. Examination revealed right-sided hemiparesis. Electrocardiogram showed sinus rhythm with anterior Q waves. Magnetic resonance imaging of the brain showed large left parietal and smaller multiple cerebral infarcts. Echocardiogram showed anterior wall and apical akinesis with a large mural thrombus. Anti-cardiolipin antibodies immunoglobulin G and immunoglobulin M were strongly positive. Coronary angiography showed dissection of the mid left anterior descending artery with normal flow down the distal vessel. He was treated conservatively with anticoagulation and secondary prevention. He was in good health when seen in clinic four months later.

**Conclusion:**

We highlight the importance of a comprehensive approach at obtaining the correct diagnosis, input of different specialities and the fact that the presence of anti-cardiolipin antibodies is associated with coronary artery dissection in a middle-aged male patient whose presentation was stroke.

## Introduction

Spontaneous coronary artery dissection (SCAD) is well described in women, especially pregnant women. We present the case of a man with SCAD which was complicated by stroke due to a left ventricular thrombus.

## Case presentation

A 56-year-old, Caucasian man presented with dysarthria and right-sided weakness to a district general hospital. There was a history of chest pain associated with sweating, nausea and vomiting four days earlier for which he had not sought medical help. He also had a history of lower limb deep vein thrombosis (DVT) four years ago. He was not taking any medications currently, and apart from his age there were no other cardiovascular risk factors.

**Figure 1:**
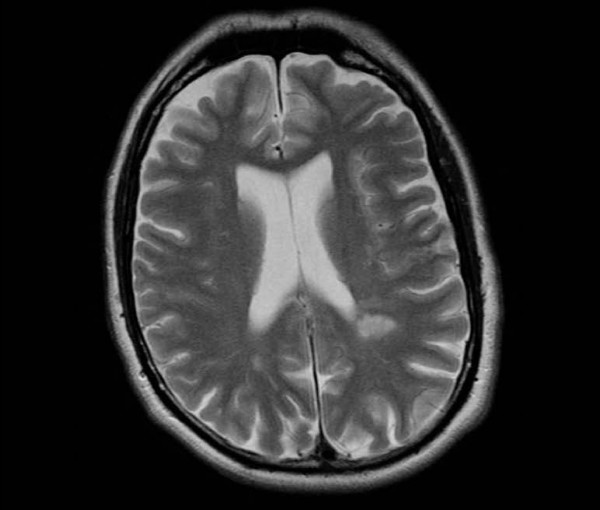
**MRI of brain showing a large left parietal and multiple smaller cerebral infarcts**.

**Figure 2:**
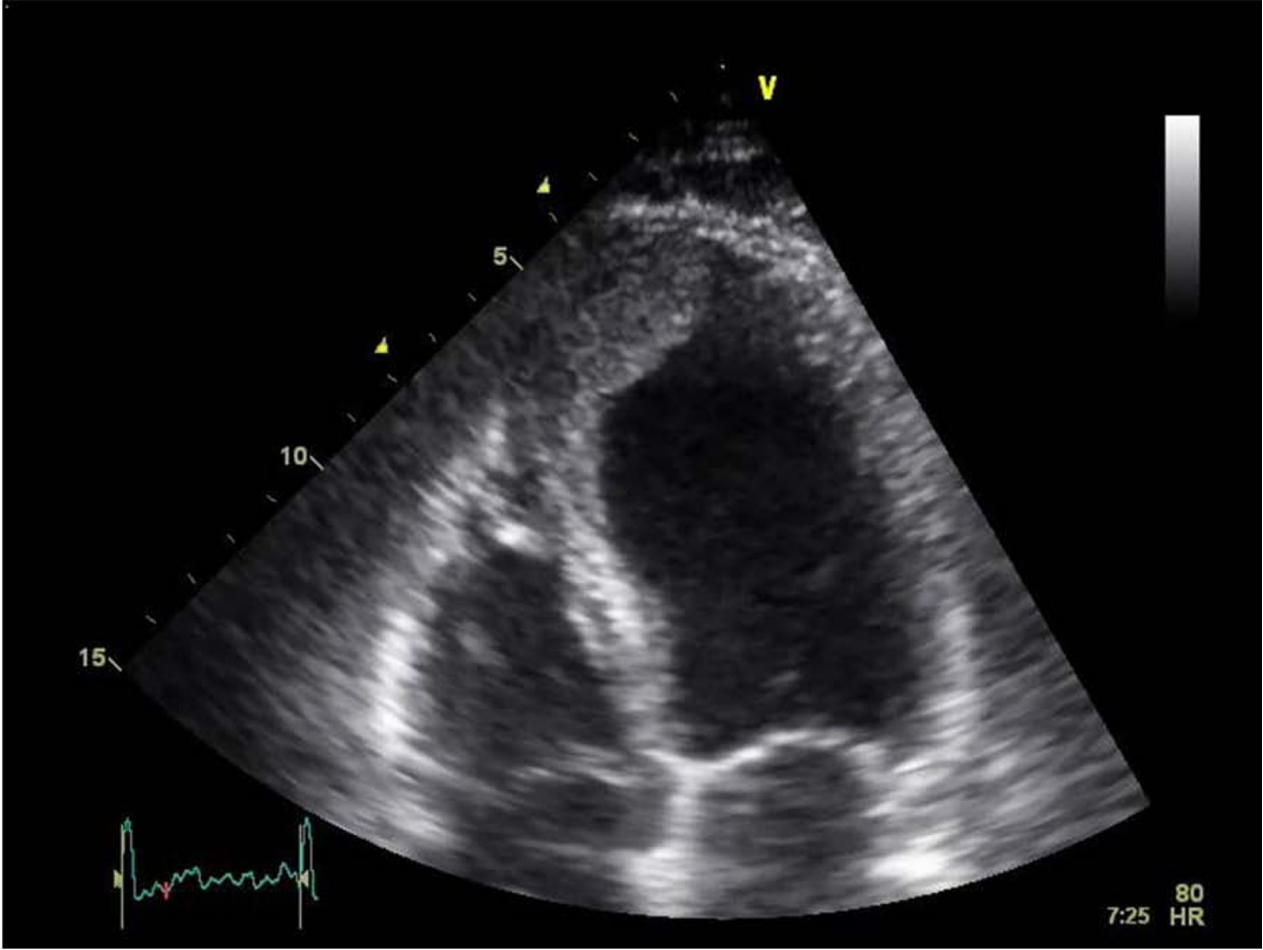
**Two-dimensional echo-4 chamber view showing left ventricular apical thrombus**.

Examination revealed right-sided weakness but nothing else of note. Electrocardiogram (ECG) showed sinus rhythm with anterior Q waves and MRI scan of the head showed a large left parietal lobe infarct and multiple smaller cerebral infarcts (Figure 1). An echocardiogram showed anterior wall and apical akinesis with a large left ventricular mural thrombus (Figure 2). Carotid Doppler measurements were all normal and blood tests revealed strongly positive anti-cardiolipin antibodies.

**Figure 3:**
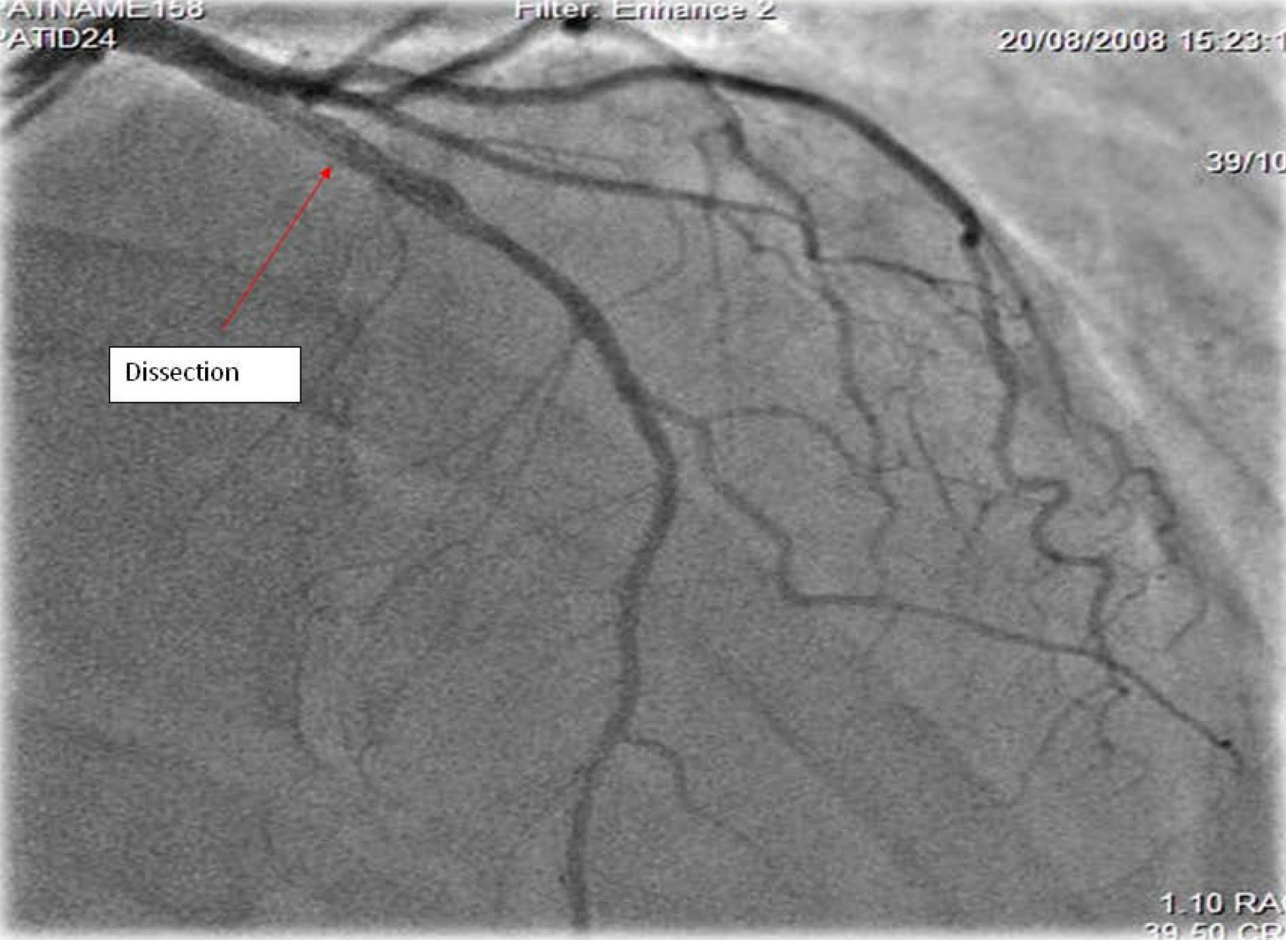
**Coronary angiography**. Posteroanterior cranial view showing contained dissection of mid left anterior descending artery (LAD).

Due to our patient's regional wall motion abnormalities, ECG changes and history, a coronary angiography was performed which showed a healed dissection of the mid left anterior descending artery with thrombolysis in myocardial infarction grade 3 (TIMI-3) flow down the distal vessel (Figure 3). The rest of the coronary arteries were all normal.

In view of the above, a diagnosis of SCAD in association with anti-cardiolipin antibodies was made. SCAD had resulted in myocardial infarction leading to the development of a left ventricular thrombus which had embolized causing a stroke, which was his presenting complaint. The patient was anticoagulated with warfarin and secondary prevention instituted with angiotensin-converting enzyme (ACE) inhibitor, statin and beta-blocker. Due to a past history of DVT, it was decided that he should continue taking warfarin for life. When reviewed in clinic four months later he had made an excellent neurological recovery and had no new symptoms. A repeat coronary angiogram 10 months after his initial presentation showed no new or progressive changes.

## Discussion

Spontaneous coronary artery dissection is a rare but potentially fatal condition, described mainly in young women, especially in the peripartum period [1]. Other associations of SCAD described in the literature are oral contraceptive use [2], antiphospholipid syndrome (APS) [3,4], connective tissue disorder [5], cocaine use [6] and physical exertion [7].

Antiphospholipid syndrome is characterized by the presence of antiphospholipid antibodies. The pathognomonic feature of this condition is recurrent thrombosis in both the arterial and venous circulations, and the possible causative mechanisms have been reviewed recently [8]. With particular reference to SCAD, recent evidence suggests a widespread endothelial dysfunction in APS [9]. Coronary endothelial dysfunction could therefore play a major role in the pathogenesis of SCAD along with other factors such as plaque or vasa vasorum rupture, localized vasculitis with eosinophilic infiltration, and increased shear stress. The preponderance of SCAD in pregnancy is also thought to be due to the high circulating levels of oestrogen and progesterone. Therefore a high degree of suspicion about APS and SCAD is necessary when dealing with pregnant or young women presenting with chest pains and ECG changes. While strokes secondary to SCAD are reported [10,11], albeit extremely rarely and in younger subjects, they have not been reported, to the best of our knowledge, in a middle-aged man in association with anti-cardiolipin antibodies.

Treatment of SCAD, although lacking in consensus, depends on the clinical situation. If the patient is asymptomatic and stable, conservative management is advised, as in the case of our patient. Successful revascularization in acutely unwell patients, in the form of percutaneous coronary intervention [12] and coronary artery bypass grafting [13] have been described in the literature. Thrombolysis can be devastating if administered in the presence of SCAD [14,15]. The cornerstone of treatment to prevent recurrent thrombosis is anticoagulation.

## Conclusion

Although carotid artery disease is the major culprit in ischemic strokes, it is important to exclude an embolic phenomenon in younger patients. Left ventricular thrombus is one of the potential embolic causes. Our patient's stroke was due to embolization from a ventricular thrombus which was in turn caused by a coronary artery dissection in the presence of anti-cardiolipin antibodies, an association rarely described in middle-aged men. It is therefore important to have a comprehensive approach to diagnosis and evidence-based management in such patients, which in turn highlights the importance of multi-disciplinary teams working together.

## Consent

Written informed consent was obtained from the patient for publication of this case report and accompanying images. A copy of the written consent is available for review by the Editor-in-Chief of this journal.

## Competing interests

The authors declare that they have no competing interests.

## Authors' contributions

NSR is the principal author who performed the literature search and drafted the case report, FL helped in literature search and NS is the consultant in charge of the patient's clinical care. NS also refined the manuscript. All authors have read and approved the final manuscript.
